# Retrospective Analysis of Hearing Outcomes of Cochlear Implantation in Patients with Deafness Due to Congenital CMV Infection

**DOI:** 10.3390/jcm14082575

**Published:** 2025-04-09

**Authors:** Natalia Zajdel, Oskar Rosiak, Nikodem Pietrzak, Maciej Skalski, Wiesław Konopka

**Affiliations:** Department of Otolaryngology, Polish Mother’s Memorial Hospital Research Institute, 93-338 Lodz, Poland; oskar.rosiak@iczmp.edu.pl (O.R.); nikodempietrzak@icloud.com (N.P.); maciej.skalski@stud.umed.lodz.pl (M.S.); wieslaw.konopka@umed.lodz.pl (W.K.)

**Keywords:** cochlear implant, congenital cytomegalovirus infection, sensorineural hearing loss, hearing outcomes

## Abstract

Cytomegalovirus (CMV) infection in pregnant women is one of the most common causes of congenital infection in children. It is often asymptomatic but can lead to serious complications, including progressive sensorineural hearing loss. Profound hearing loss is an indication for cochlear implantation (CI). Electrode impedance and neural response telemetry (NRT) thresholds can be measured to confirm correct electrode placement and speech processor programming. **Background/Objectives**: The aim of the study is to evaluate the hearing outcome of children with profound sensorineural hearing loss or deafness due to cCMV infection after CI compared to a control group of children born with other causes of congenital hearing loss and to identify prognostic factors predicting the outcome of patients with hearing loss due to cCMV infection after CI. **Methods**: A retrospective study was conducted in patients implanted between 2016 and 2023 at the Department of Otolaryngology of the Institute of the Polish Mother’s Memorial Hospital Research Institute in Łódź. Pre- and postoperative hearing levels, electrode impedance and neural response telemetry (NRT) thresholds were compared. The degree of pre-implantation hearing loss was assessed by the level of the recorded V-wave in the ABR test. Post-implantation hearing assessment was based on the last available free-field tonal audiometry measurement. Impedance measurements were included: intraoperative, 1, 6, 12 months after CI, respectively, and NRT thresholds. **Results**: The final analysis included 84 patients with profound sensorineural hearing loss and complete audiological follow-up data: 13 patients with congenital CMV (cCMV) infection and 71 patients with other causes of deafnes. The analysis included 175 implanted ears: 17 in the CMV group and 158 in the control group. The age at implantation ranged from 1 to 11 years in the CMV and from 1 to 13 years in the control group. Mean preoperative hearing thresholds were 94.54 dB in the CMV group and 97.04 dB in the control group. At the most recent postoperative evaluation, mean thresholds improved to 33.83 dB and 36.42 dB, respectively. No statistically significant differences were observed between the groups. Mean intraoperative NRT values were 79.74 in the CMV group and 86.90 in the non-CMV group. Final NRT values were 129.77 and 130.76, respectively. Mean impedance values measured intraoperatively and at 1, 6 and 12 months postoperatively were 11.09 kOhm, 13.40 kOhm, 8.35 kOhm and 8.25 kOhm in the CMV group; and 12.28 kOhm, 14.06 kOhm, 9.60 kOhm and 8.00 kOhm in the control group, respectively. **Conclusions**: CI in children with deafness caused by cCMV infection is an effective treatment option. Initial electrical impedance values of the electrodes increase after implant activation and decrease in subsequent months of follow-up, suggesting the absence of active adhesion processes in the cochlea.

## 1. Introduction

Congenital hearing loss is one of the most prevalent sensory disorders in children. It can be caused by environmental factors, prenatal factors, congenital infections and genetic factors, which are responsible for most cases [1,2]. Defining the aetiology of congenital hearing loss is important for making appropriate therapeutic decisions, but for a significant number of infants with hearing loss a definitive cause cannot be identified [1]. Early diagnosis is possible through neonatal hearing screening programmes. Treatment options depend on the type of hearing loss (conductive or sensorineural) and include surgical treatment, implantable or non-implantable hearing aids, or recently, targeted gene therapy [1,3,4]. Prompt intervention is necessary to prevent delayed speech and language development and to avoid a negative impact on the child’s social and emotional development [1].

Human cytomegalovirus (CMV), a DNA virus belonging to the Herpesviridae family, is considered one of the most common congenital infections [5]. Vertical transmission of CMV may lead to intrauterine growth retardation, low birth weight, cognitive impairment, motor and visual deficits, hepatosplenomegaly, multiple haematological abnormalities, skin lesions in newborns and—the most significant—neurodevelopmental disorders, including sensorineural hearing loss [4,6,7,8]. CMV is the leading cause of nongenetic sensorineural hearing loss (SNHL), responsible for up to 10% of cases in children born to infected mothers, including both unilateral and bilateral SNHL [3,4,6]. While the incidence of SNHL is similar between primary and non-primary maternal infections, primary infections tend to result in more severe and bilateral SNHL [6]. Diagnosis of cCMV is possible within three weeks of birth by detecting CMV DNA in urine, saliva or blood. However, due to the lack of routine CMV screening, absence of clinical symptoms in many cases and the difficulty in collecting adequate samples at 3 weeks after birth, a significant number of infants may not be diagnosed in time [4]. In most children with cCMV, hearing loss is severe to profound, progressive with a variable nature and a late onset, which is why it may not be detected by universal newborn hearing screening (NHS) [5,6]. Thus, children diagnosed with cCMV require monitoring of hearing function with regular audiological evaluations until at least 6–8 years of age [3,4,6,9]. The prognosis of patients with cCMV infection is variable. Although about 85–90% of infants with cCMV are asymptomatic, 10–15% of them will develop SNHL. Among children with the symptomatic form, it will appear in up to 40–60% [6,7]. Symptomatic cCMV is typically associated with more severe and bilateral SNHL [3]. There is a high risk of SNHL and developmental delay in children with central nervous system disease.

In order to determine the appropriate treatment, early diagnosis of CMV infection is important. Symptomatic congenital CMV infection treated with intravenous antiviral therapy can reduce the severity of complications, including hearing loss [10,11,12]. Moreover, prompt diagnosis can provide early aural rehabilitation [10]. Early diagnosis and prompt intervention are crucial for optimal speech and language development, as well as for minimizing the social and emotional impact of hearing loss [3,5].

In cases of profound SNHL, cochlear implantation (CI) is a long-established treatment option [13]. Children with cCMV are at risk of developing profound SNHL either at birth or during childhood and may benefit from CI. It is estimated that 2% of children with asymptomatic cCMV have a hearing loss significant enough to be considered a candidate for a cochlear implant [3]. The outcomes are the best in infants with limited duration of auditory deprivation, although the results are still inconsistent, particularly in children with significant neurocognitive deficits [5,14,15]. This form of treatment requires many years of postoperative rehabilitation; so awareness, proper motivation and support for the patient is important. Each candidate undergoes a multi-stage qualification that verifies the potential benefits of the planned surgery [13].

Auditory nerve and brainstem responses provide a measure of hearing loss before implantation [5]. After successful implantation, electrophysiological (objective) and behavioural (subjective) parameters are used to set the parameters of cochlear implant speech processors [13] and monitor the progress of hearing rehabilitation. Other objective parameters, such as the impedance of the electrodes, can be used to monitor for possible fibrotic changes within the cochlea. Electrical current stimulation at the electrode can be manipulated to provide a comfortable hearing level according to objective parameters derived directly from the measurements conducted from the implant electrodes.

While studies have demonstrated the effectiveness of cochlear implants in improving hearing outcomes for cCMV patients [16], there is a paucity of research specifically examining the correlation between electrode impedance and cochlear fibrosis in this population. Given that cCMV infection can trigger inflammatory responses within the cochlea, understanding impedance trends in cCMV patients is essential, as cochlear fibrosis could potentially influence the long-term success of CI. The aim of this study was to provide longitudinal impedance data to account for the initial and later inflamatory response in the cochlea.

## 2. Materials and Methods

A retrospective study was conducted in patients implanted between 2016 and 2023 at the Department of Otolaryngology of the Institute of the Polish Mother’s Memorial Hospital Research Institute in Łódź.

As this study was retrospective in nature, ethics committee approval was not required in accordance with local regulations. Patients provided informed consent for their data to be analyzed anonymously, and all patient information was anonymized.

Pre- and postoperative hearing levels, electrode impedance and neural response telemetry (NRT) thresholds were compared. The degree of pre-implantation hearing loss was assessed by the level of the recorded V-wave in the ABR test at 500 Hz, 1 kHz, 2 kHz and 4 kHz. Post-implantation hearing assessment was based on the last available free-field tonal audiometry measurement. Impedance measurements were included: intraoperative, 1, 6, 12 months after CI, respectively, and NRT thresholds. The diagnosis of congenital CMV infection in the patients included in our study was made by the neonatal centre providing postnatal care to the patient. Patients were then referred to our centre due to hearing loss. Detailed data is not available. Speech recognition scores were only available for a minority of patients and for this reason were not included in the analysis.

All patients included in the study had bilateral, prelingual, profound sensorineural hearing loss, as confirmed by ABR and documented clinical data. In all cCMV cases, hearing loss was identified in early childhood, following failed newborn hearing screening or during routine paediatric assessment.

Continuous variables were summarized using mean and standard deviation (SD). The Shapiro–Wilk test was used to assess the normal distribution of continuous variables. A comparison between the groups was conducted using the Mann–Whitney U test for non-normally distributed variables. The level of significance used for all analyses was 2-tailed and set at *p* < 0.05. Statistical analysis was performed using STATISTICA software (Version 13.1, Dell).

### 2.1. Electrophysiological Parameters

#### 2.1.1. Electrode Impedance

Electrode impedance is measured both intraoperatively and postoperatively. Although it is rarely used to set up speech processors, it provides important information about the electrode environment, as it is sensitive to local fibrosis, bending, changing position, extrusion or electrode damage. The impedance of the most basal electrodes increases during the first postoperative months and later stabilizes, but remains higher than the impedance of the middle and apical electrodes, which show decreased impedance values between the 1st and 6th postoperative months and a stabilization in the later course [12].

#### 2.1.2. Neural Response Telemetry (NRT)

NRT is the lowest level of electrical stimulation evoking a cochlear nerve response. It may be used to check the integrity of the electrode chain when it is inserted in the cochlea [15]. It tends to decrease during the first 3 months after treatment and then reach a plateau. Due to its stable values, it becomes a valuable tool for setting thresholds of behavioural parameters [12].

### 2.2. Behavioural Parameters

#### 2.2.1. T (Threshold Level)

T level is defined as the quietest sound the patient is able to hear for a specific electrode.

#### 2.2.2. C (Comfort Level)

Level C is defined as the loudest sound that does not cause uncomfortable sensations for the patient.

These parameters can be measured by using electrophysiological parameters and by monitoring the patient’s response to the sounds delivered to each electrode. In the initial post-implantation period, the difference between T and C values is small and increases as rehabilitation progresses and the auditory pathway adapts to the cochlear implant, resulting in improved hearing quality.

Once the speech processor has been fitted, it is important to estimate the effectiveness of the cochlear implant. Free field audiometry (FFA) with and without the speech processor is performed to assess the benefit of implantation. It measures the patient’s thresholds with the processor and shows any improvement compared to hearing without the processor [17].

To assess electrode function and cochlear response, impedance values were analyzed intraoperatively and during follow-up at 1, 6 and 12 months. The analysis included mean impedance values across all electrodes as well as by their position within the cochlea. This allowed us to evaluate potential regional differences that might indicate localized fibrosis or variability in the cochlear environment [18,19].

## 3. Results

A total of 84 patients (47 males, 37 females) with profound sensorineural hearing loss and complete audiological follow-up data were included in the final analysis. Among them, 13 patients were diagnosed with congenital CMV (cCMV) infection and 71 patients had other confirmed or presumed causes of deafness and constituted the control group (Table 1). The analysis included 175 implanted ears: 17 in the CMV group and 158 in the control group. A total of 40 patients received bilateral implants. In one case, only one side was included due to incomplete measurement records for the contralateral implant. In the CMV group, 82.35% (*n* = 14/17) received a Nucleus CI612, 5.88% (*n* = 1/17) CI632, and 5.88% (*n* = 1/17) CI512. In the control group, 52.43% (*n* = 54/103) received CI612, 22.33% (*n* = 23/103) CI512, 18.45% (*n* = 19/103) CI522 and 3.88% (*n* = 4/103) CI632.

The age at implantation ranged from 1 to 11 years in the CMV group (*n* = 17, mean = 3.98 years, SD = 2.66) and from 1 to 13 years in the control group (*n* = 158). In the CMV group, 1 patient (5.9%) was implanted before the age of 2 years, while 16 patients (94.1%) were 2 years or older. In the control group, 13 patients (8.2%) were younger than 2 years and 145 (91.8%) were 2 years or older at the time of implantation. Of these, postoperative hearing threshold data (PTApost) were available for 100 patients in the ≥2 years subgroup.

**Figure 1:**
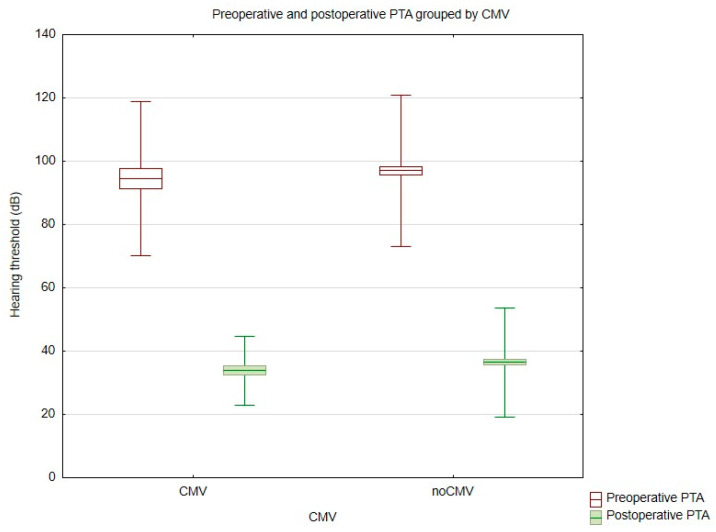
Comparison of pre- and postoperative values of PTA in cCMV and non-cCMV patients.

Mean preoperative hearing thresholds were 94.54 dB (SD = 12.16) in the CMV group and 97.04 dB (SD = 11.98) in the control group. At the most recent postoperative evaluation, mean thresholds improved to 33.83 dB (SD = 5.44) and 36.42 dB (SD = 8.59), respectively (Figure 1). No statistically significant differences were observed between the groups.

As only one patient in the CMV group was implanted before the age of 2 years, no statistical comparisons were performed between <2 and ≥2 year subgroups in this group.

**Figure 2:**
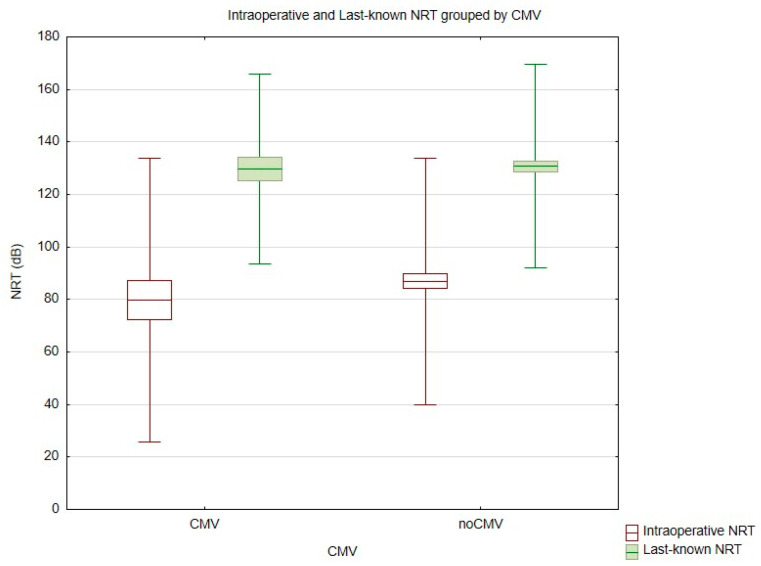
Comparison of intraoperative and last-known values of NRT in cCMV and non-cCMV patients. NRT (Neural Response Telemetry).

Mean intraoperative NRT values were 79.74 (SD = 27.05) in the CMV group and 86.90 (SD = 23.50) in the non-CMV group. Final NRT values were 129.77 (SD = 18.11) and 130.76 (SD = 19.43), respectively (Figure 2).

Mean impedance values measured intraoperatively and at 1, 6 and 12 months postoperatively were 11.09 kOhm (SD = 2.61), 13.40 kOhm (SD = 1.69), 8.35 kOhm (SD = 1.18) and 8.25 kOhm (SD = 0.95) in the CMV group; and 12.28 kOhm (SD = 8.21), 14.06 kOhm (SD = 8.37), 9.60 kOhm (SD = 6.40) and 8.00 kOhm (SD = 1.13) in the control group, respectively. These values represent an average across all angular ranges of the electrode.

**Figure 3:**
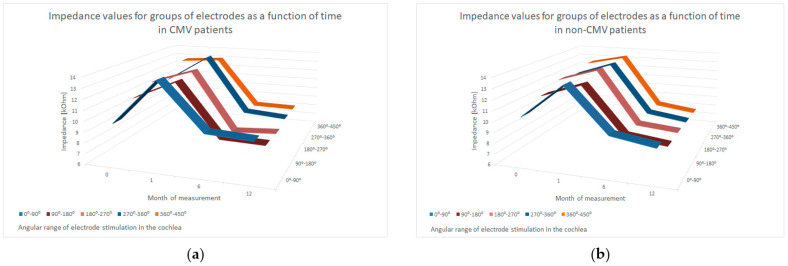
Impedance values for groups of electrodes as a function of time. (**a**) cCMV patients, (**b**) non-cCMV patients.

Additionally, we analyzed impedance evolution across five angular insertion ranges within the cochlea. As shown in Figure 3, no statistically significant differences were found, suggesting that no specific region was more prone to increased impedance or suspected fibrosis. All analyzed parameters are summarized in Table 2.

## 4. Discussion

In this study, we showed that hearing outcomes in 13 patients who underwent cochlear implantation (CI) for sensorineural hearing loss (SNHL) caused by congenital CMV infection were not significantly different from those in the control group of 71 patients with other causes of congenital deafness. These findings confirm the effectiveness of CI in improving auditory thresholds across different aetiologies. The exact mechanism of hearing loss due to cCMV infection is still uncertain, with two pathophysiological hypotheses proposed. The first suggests direct viral damage to spiral ganglion neurons or hair cells, potentially through damage to Reissner’s membrane or the vascular striatum, and disruption of potassium homeostasis in the organ of Corti, contributing to sensorineural hearing loss. The second theory involves immunological damage caused by the host immune system’s response to CMV-infected cells [1,3].

Positive hearing outcomes after CI implantation in cCMV children were also observed in other studies. According to Yamazaki, hearing thresholds in both CMV and genetic CI were almost identical [15]. Bolduc has shown that the audiological results of implantation in patients with cCMV are satisfying for proper functioning [12]. Several other papers report that not only pure tone thresholds but also speech perception and language production can be improved after CI, even in children with deafness associated with cCMV infection [10,16,20,21].

Even when CI was effective, children with cCMV hearing loss show lower levels of speech comprehension and oral language development than those with hearing loss due to other causes, which may be influenced by coexisting neurodevelopmental disorders [10,15,16,20,21,22]. These children are considered to be more likely to experience later complications such as learning difficulties [10,16]. Nevertheless, SNHL associated with developmental delay should not be a contraindication to implantation [21]. Lee et al. showed that patients with cCMV-related severe to profound SNHL, even with CNS disabilities, such as cognitive delay, also demonstrated significant gains in hearing and speech, highlighting the importance of early rehabilitation after surgery [11]. According to Yamazaki, a younger age at the time of CI leads to better speech development [15]. Thus, children with multiple disorders should be offered informed consent, early CI and comprehensive rehabilitation, as this can successfully improve their outcomes, even in the presence of coexisting CNS abnormalities [11,16,21]. For this reason, it is crucial to identify such conditions before surgery and to inform patients or their caregivers about the risk of limited benefits from Cl and the need for intensive postoperative rehabilitation according to the level of development [16,21].

Elevated impedance values can indicate the formation of fibrotic tissue around the electrodes, which may impede electrical signal transmission and affect auditory performance [23]. The proper function of a cochlear implant can be confirmed by monitoring the impedance values for each electrode. An increase in the impedance values may indicate fibrous tissue formation around the electrode, which may be a response to foreign body inflammation or penetration of bone dust or blood into the perilymph and may cause the available voltage between the electrodes to be insufficient to generate a stimulus of the required amplitude to stimulate the auditory nerve endings. On the other hand, high voltage stimulation is damaging to the tissue and the electrode [24,25].

The initial electrode impedance values in the patients included in our retrospective study increase after implant activation and decrease in the following months. These changes may be explained by the fact that electrical stimulation causes regression of fibrotic lesions developed in the inner ear after implantation and prevents their recurrence [25].

Our results are compatible with those of other authors. Zadrożniak et al. observed an increase in electrical impedance values when measured approximately 1 month after cochlear implant surgery, during speech processor activation, compared to intraoperative measurements. In subsequent measurements 1 month, 6 months and 12 months after speech processor activation, respectively, impedance values were statistically lower for all electrodes [25]. According to Busby et al., the lowest impedances were found intraoperatively, then the values increased and the highest impedances were found during the first session, then decreased until one week after fitting [26]. Similar observations were also described by other authors [27,28].

In our study, we found no statistically significant differences between the groups, which may suggest that there is no increased tendency for peri-cochlear fibrosis in patients with cCMV compared to patients with other causes of deafness.

Our study has several limitations. The main drawback was its retrospective design: children were not routinely screened for cCMV infection, the cCMV infection status was known upon referral to the implantation centre, so the number of confirmed cCMV-infected cochlear implant recipients was limited; children with neurological disorders were not identified. It is important to note that only one patient in the CMV group was implanted before the age of 2 years. Therefore, the results for this group should be interpreted with caution, and no meaningful statistical conclusions can be drawn. Although the data were included for completeness, no meaningful conclusions can be drawn regarding early implantation outcomes in this population. This imbalance should be taken into consideration when interpreting the results. We did not have access to CT or MRI data to directly confirm cochlear fibrosis. Therefore, inferences were based solely on impedance trends, which is a limitation of this study. Moreover, we did not obtain speech recognition scores, which is an important benefit of hearing implantation for proper functioning in society.

## 5. Conclusions

CI in children with deafness caused by cCMV infection is an effective treatment option. Initial electrical impedance values of the electrodes increase after implant activation and decrease in subsequent months of follow-up, suggesting the absence of active adhesion processes in the cochlea.

## Figures and Tables

**Table 1:** Causes of hearing loss in non-CMV patients.

Cause of Hearing Loss	Number of Patients
Genetic, non-syndromic	3
Genetic, syndromic (ADOA plus syndrome)	2
Genetic, syndromic (Berdon syndrome)	1
Gentamicin treatment	4
Complications following treatment of cardiac disorders	17
Idiopathic/cause not documented	42

**Table 2:** Mean values of hearing thresholds and electrophysiological parameters in the CMV and non-CMV group.

		CMV	Controls	*p*=
Preoperative hearing thresholds		94.54 dB SD 12.16 95%CI [88.62; 100.45]	97.04 dB SD 11.98 95%CI [93.05; 101.03]	0.48
Postoperative hearing thresholds		33.83 dB SD 5.44 95%CI [30.83; 36.83]	36,42 dB SD 8,59 95%CI [33.00; 39.84]	0.34
Impedance (mean of all electrodes)	intraoperative	11.09 kOhm SD 2.61 95%CI [9.89; 12.29]	12.28 kOhm SD 8.21 95%CI [9.05; 15.51]	0.54
	after 1 month	13.40 kOhm SD 1.69 95%CI [12.44; 14.36]	14.06 kOhm SD 8.37 95%CI [10.80; 17.32]	0.87
	after 6 months	8.35 kOhm SD 1.18 95%CI [7.61; 9.09]	9.60 kOhm SD 6.40 95%CI [6.83; 12.37]	0.61
	after 12 months	8.25 kOhm SD 0.95 95%CI [7.70; 8,80]	8.00 kOhm SD 1.13 95%CI [7.38; 8.62]	0.46
NRT (intraoperative)		79.74 SD 27.05 95%CI [67.11; 92.37]	86.90 SD 23.50 95%CI [77.97; 95.83]	0.37
NRT (last-known)		129.77 SD 18.11 95%CI [121.06; 134.48]	130.76 SD 19.43 95%CI [122.54; 138.98]	0.98

NRT (Neural Response Telemetry).

## Data Availability

The datasets generated and/or analyzed during the current study are not publicly available due the state of the hospital but are available from the corresponding author on reasonable request.
